# Evaluation of strain in mandibular denture-supporting area in three different occlusal schemes during jaw movements

**DOI:** 10.15171/joddd.2018.004

**Published:** 2018-03-14

**Authors:** Ali Hafezeqoran, Roodabeh Koodaryan, Seyed Gholamreza Noorazar, Masoud Hajialilue-Bonab, Mehran Hassanzadeh, Neda Yasamineh

**Affiliations:** ^1^Dental and Periodontal Research Center, Tabriz University of Medical Sciences, Tabriz, Iran; ^2^Research center of Psychiatry and Behavioral Sciences, Tabriz University of Medical Sciences, Tabriz, Iran; ^3^Department of Civil Engineering, University of Tabriz, Iran

**Keywords:** Bilateral balanced, buccal shelf, distolingual, functional stress, lingualized occlusion, monoplane

## Abstract

***Background.*** Various occlusal schemes have been introduced over the years to enhance the stability, comfort, beauty and function of complete denture, of which lingualized, bilateral balanced and monoplane occlusions are the most recommended. The aim of this study was to compare the strain in mandibular denture-supporting structures in three different occlusal schemes.

***Methods.*** Two mandibular and maxillary models were simulated using epoxy resin, and strain gauges were embedded on each side of the mandible in mental foramen, buccal shelf and distolingual area. Strain values were measured in three occlusal schemes at centric occlusion protrusive and lateral movements. Data were analyzed with one-way and three-way ANOVA, followed by post-hoc Tukey tests. The significant level was set at 0.05.

***Results.*** The mean strain in denture-supporting area was lower in monoplane occlusion than the two other occlusal schemes, and the mean of values in the buccal shelf was higher than that of mental foramen and distolingual area. In all the three occlusal schemes, the mean strain values on the working side were higher than those on the non-working side during eccentric movements.

***Conclusion.*** Monoplane occlusal scheme imposed lower strain on denture-supporting area, with the buccal shelf being the primary strain-bearing area to tolerate more pressure than the rest of the denture-supporting areas. In terms of strain distribu-tion scheme, in all the three occlusal schemes, the working side received more strain than the non-working side during eccen-tric movements.

## Introduction

Complete dentures are made to restore function of natural teeth. An ideal arrangement of teeth able to provide maximize stability, comfort, esthetics and function has been the subject of many investigations over the years and are still continuing.^1^ Therefore, selection of occlusal schemes is an important factor in the fabrication of complete dentures.^2^ Among the different occlusal schemes, lingualized, bilaterally balanced and monoplane occlusions have been used mostly in denture construction.^2^ In fact, transmission of masticatory forces to the underneath the edentulous ridge is influenced by the size, shape and occlusal scheme of the denture posterior teeth.^3^ Researchers have also paid considerable attention to masticatory efficacy,^4-6^ patient satisfaction^7,8^ and measurement of forces exerted by artificial teeth during mastication.^9-12^ Understanding these forces and the distribution scheme of stress on the bone beneath complete dentures are the most important priorities during fabrication of complete dentures.

Alveolar ridge atrophy poses a clinical challenge toward the fabrication of successful prosthesis. Resorption of mandibular ridge results in unstable and non-retentive dentures associated with pain and discomfort for edentulous patients.^13^

Complete dentures are known as the causative factor of mandibular ridge resorption during function. Ridge atrophy can arise from compressive forces generated in dentures that exceed physiological tolerance of the underlying bone.^9^ Few studies have compared the distribution of strain between lingualized, bilaterally balanced and monoplane occlusal schemes. For example, Swoope and Kydd^10^ showed that reduction of cusp angle of posterior artificial teeth leads to a decrease in pressure on complete denture bases. Madalli et al^3^ compared the pressure on the denture-supporting area in different occlusal schemes and concluded that the stress on the denture-supporting area was lower in monoplane occlusal scheme than the anatomical and lingualized schemes. Sharry et al^14^ showed on dry skull that more stress is exerted on the bone by anatomical teeth of the denture compared to the zero-degree teeth. This study was undertaken to investigate factors affecting the distribution of strain in different occlusal schemes in the edentulous ridge.

## Methods

### Preparation of models

A set of alginate maxillary and mandibular impressions was taken from randomly chosen edentulous patients with mandibular residual ridge atrophy. The statuses of atrophied ridge were: loss of sulcus width and depth, displacement of the muscle attachments closer to the ridge, loss of VDO and mental foramen close to the top of the residual ridge.^15^ The impressions were poured using a type III die stone (Mold Stone, Pars Dandan, Iran). Custom trays were fabricated by fitting 2 layers of wax over the cast to provide a 2-mm thickness of impression material.

**Figure 1:**
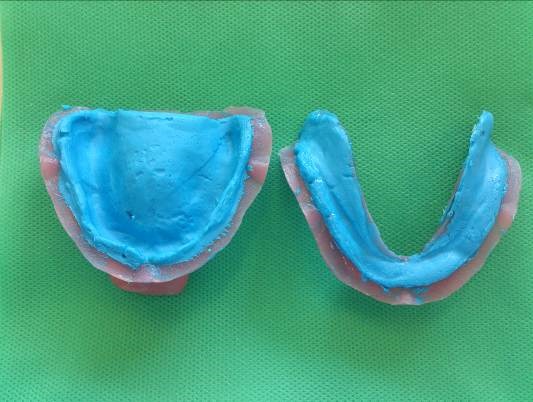
Monophasic impression with custom tray.

A 2-mm-thick custom tray with visible light-cured resin (Mega-Light Tray, Mega Dental, and GmbH, Germany) was prepared by embedding resin in anterior and posterior stops of the casts and placing a 2-mm spacer. Then monophasic impression (Panasil Monophase Medium, Kettenbach, GmbH, Germany) was made (Figure 1).

**Figure 2:**
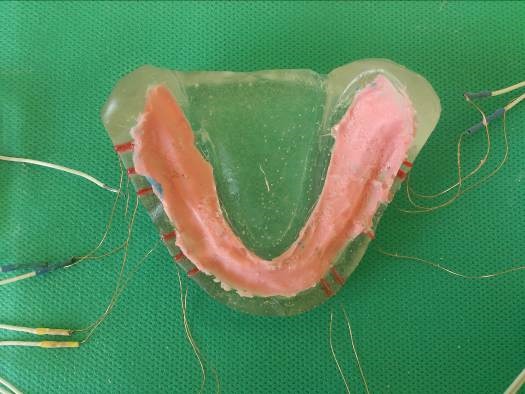
Study model (epoxy resin) with Gingimask.

The thickness of the ridge was reduced to about 2 mm for the maxillary and 1.5 mm for the mandibular edentulous ridge, corresponding to the desired mucosal thickness.^16^ An impression was made to obtain an epoxy resin (EPON^TM^828, Hardner F 205; PMP Company, Tehran, Iran) model on which strain gauges (KFG-1-120-C1-11L1M2R; KYOWA Electronic Instruments, Tokyo, Japan) were installed. The oral mucosa was simulated with silicon Gingifast (Zhermack, A-silicone for gingival mask, Italy) by using the first monophasic impression with custom tray (Figures 1 and 2).

**Figure 3:**
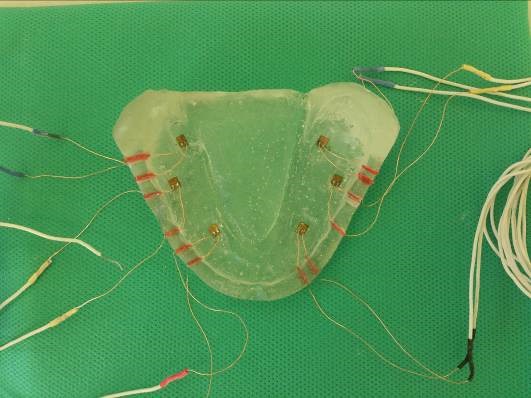
Study model (epoxy resin) with strain gauges.

Therefore, six strain gauges (2×2.5×0.1) were positioned to record the strain in the bone adjacent to mental foramina, buccal shelves and distolingual areas bilaterally. The strain gauges were bonded with quick-setting cyanoacrylate adhesive (QUICK STAR, Instant Super Glue, Zhejiang, China) in their respective locations (Figure 3). The resin casts with Gingifast (Zhermack, A-silicone for gingival mask, Italy) were the final model of maxilla and mandible.

The final model was molded three times with monophasic silicone (Panasil Monophase Medium, Kettenbach, GmbH, Germany) and the custom tray with visible light-cured resin (Mega-Light Tray, Mega Dental, and GmbH, Germany). Then the impressions were cast with dental stone type III (Mold Stone, Pars Dandan, Iran). A total of 3 pairs of stone casts were produced from the maxilla and mandible. The trial bases were made for setting up the teeth on the stony casts.

### Dental arrangement in three different occlusal schemes

Three test groups of complete denture prostheses with different occlusal schemes were fabricated on the stony model. The teeth were arranged in 3 groups:

Group 1. Monoplane occlusal scheme;8 monoplane acrylic teeth(I_11_ M_3_ B Star, Ideal Makoo, Iran). The articulator was set with the sagittal and lateral path inclinations at 0°.^15^

Group 2. Lingualized occlusal scheme;8 lingualized acrylic teeth(I_11_ N_3_ Nano Glass, Ideal Makoo, Iran). The articulator was set with the sagittal and lateral path inclinations at 25° and 15°, respectively.^15^

Group 3. Bilaterally balanced occlusal scheme;8 anatomic acrylic teeth (I_11_ N_3_ B Star, Ideal Makoo, Iran). The articulator was set with the sagittal and lateral path inclinations at 25° and 15°, respectively.^15^

Finally, all the groups were waxed up and processed with heat-cured acrylic resin (Vertex, Conventional Heat Curing Denture Base Material, The Netherlands) according to the manufacturer’s recommendations. The prepared dentures were remounted and laboratory adjustments8 were performed for correction of processing changes and reclaiming of VDO. Finally, the occlusions were balanced through selective abrasion in all the three occlusal schemes.^15^ Then the dentures were separated from their casts by carefully cutting them and placed on the prepared resin models. In order to mount the resin models similar to each of the occlusal groups and to record the maxillary position, an index was prepared from silicone material (Dental Speedex Putty Coltene Whaledent, United states) in each group before arrangement of the teeth. To be ensured of the similarity of the position of maxilla we used silicone index (Dental Speedex Putty Coltene Whaledent, United states) and for similarity of the position of maxilla compared to mandible in the different occlusal groups and resin models, the amounts of overbite and overjet were defined, and the midline and Class I dental relationship were our criteria.^3^ The resin model was mounted on a semi-adjustable articulator [HANAU, NON ARCON, Brazil].

### Load application

**Figure 4:**
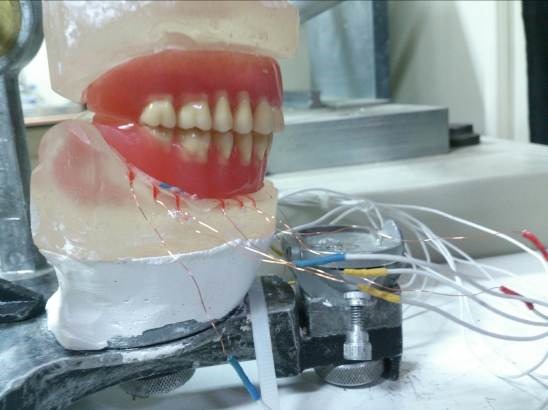
Connecting the articulator to mechanical device.

To exert force, the articulator was attached to a mechanical device which could simulate jaw movements. It can induce the articulator to make lateral and protrusive movements at the range of 3 mm, and simultaneously, can exert a perpendicular force of 110 N on the occlusal surfaces of the teeth (Figure 4).

Strain exerted on the edentulous ridge was recorded in 1) centric occlusion, 2) eccentric protrusive movement as much as 3 mm, and 3) eccentric lateral movement as much as 3 mm under the force of 110 N exerted by the jaw movements simulator to the center of the articulator. This force is equivalent to the masticatory forces in edentulous patients with complete dentures**.3** Each experiment was repeated four times for any of the above-mentioned positions and the resultant strains were amplified and recorded by signals received from sensors through a six-channel electronic monitor.^3^ Data were analyzed with one-way and three-way ANOVA followed by post-hoc Tukey tests at the 0.05 significance level.

## Results

The results of Table 1 shows that the mean strain in anatomical occlusal scheme was higher and in monoplane occlusal scheme was lower than the two other schemes.

**Table 1:** Comparison of mean strain values in three occlusal schemes with post-hoc tests (με)

**(I) Occlusion**	**(J) Occlusion**	**Mean Difference (I-J)**	**Std. Error**	**P-value**
**Anatomic**	**Lingualized**	1.5818	.39197	<.001
**Anatomic**	**Monoplane**	4.2777	.39825	<.001
**Lingualized**	**Monoplane**	2.6959	.39825	<.001

There was a significant difference between the mean strain between the three studied areas (P<0.001).

The results of Table 2 shows that the mean strain values were higher in the buccal shelf than mental foramen and distolingual areas, but there was no statistically significant difference between mental foramen and distolingual area (Table 2).

**Table 2:** Comparison of mean strain values in three studied areas with post-hoc tests (με)

**(I) Area**	**(J) Area**	**Mean Difference (I-J)**	**Std. Error**	**P-value**
**Distolingual**	**Buccal shelf**	-3.0227	.39805	<.001
**Distolingual**	**Mental foramen**	-.6845	.39502	. 194
**Buccal shelf**	**Mental foramen**	2.3381	.39502	<.001

The effect of the studied movements on the mean strain variable was not the same in different occlusal schemes (P<0.001).

Table 3 shows that in the anatomic occlusal scheme, the mean strain value of working side was higher than that in the protrusive movement, centric occlusion and non-working side (P<0.001, P=0.004 and P<0.001, respectively). Moreover, mean strain value was higher in centric occlusion than in protrusive movement (P<0.001).

**Table 3:** Comparison of the mean strain values ​​in the studied movements for all the studied occlusal schemes (με)

**Occlusion**	**(I) Movement**	**(J) Movement**	**Mean Difference (I-J)**	**Std. Error**	**P-value**
**Anatomic**	Protrusive	Non-working	-3.17639	1.31332	.079
	Protrusive	working	-9.31944	1.17467	<.001
	Protrusive	Static	-5.25000	1.17467	<.001
	Non-working	working	-6.14306	1.31332	<.001
	Non-working	Static	-2.07361	1.31332	.394
	Working	Static	4.06944	1.17467	.004
**Lingualized**	Protrusive	Non-working	.24444	1.06858	.996
	Protrusive	working	-6.06111	.95577	<.001
	Protrusive	Static	2.58889	.95577	.038
	Non-working	working	-6.30556	1.06858	<.001
	Non-working	Static	2.34444	1.06858	.130
	Working	Static	8.65	.95577	<.001
**Monoplane**	Protrusive	Non-working	1.01597	.51682	.207
	Protrusive	working	-.49722	.40542	.611
	Protrusive	Static	1.61111	.40542	.001
	Non-working	working	-1.51319	.51682	.021
	Non-working	Static	.59514	.51682	.658
	Working	Static	2.10833	.40542	<.001

In lingualized occlusal scheme, the mean strain values of working side was higher than protrusive movement, centric occlusion and non-working side (P<0.001, P<0.001 and p<0.001, respectively). However, it was higher in protrusive than centric occlusion (P=0.038).

The impact on the studied areas and the mean strain variable were not the same in different movements (P<0.01). The results showed that in static posture, there was no statistically significant difference between the mean strain values in the studied areas (P>0.05). However, there was a significant difference between the mean strain values ​​in the studied areas in protrusive movements and on the working and non-working sides (P<0.05, P<0.05 and P<0.05, respectively) (Table 4).

**Table 4:** Comparison of the mean strain values ​​in the studied movements for all the studied areas (με)

**Movement**	**(I) Area**	**(J) Area**	**Mean Difference (I-J)**	**Std. Error**	**P-value**
**Protrusive**	Distolingual	Buccal shelf	-1.14444	.44095	.029
	Distolingual	Mental foramen	1.38333	.44095	.006
	Buccal shelf	Mental foramen	2.52778	.44095	<.001
**Non-working**	Distolingual	Buccal shelf	-2.21500	.72004	.009
	Distolingual	Mental foramen	-2.01250	.68939	.013
	Buccal shelf	Mental foramen	.20250	.68939	.954
**Working**	Distolingual	Buccal shelf	-6.33056	1.45167	<.001
	Distolingual	Mental foramen	-.97500	1.45167	.780
	Buccal shelf	Mental foramen	5.35556	1.45167	.001

In protrusive movement, a higher mean strain value was recorded in distolingual than mental foramen area (P=0.006).

On the non-working side, the mean strain values in mental foramen was higher than the distolingual area (P=0.013).

The results of this study showed statistically significant differences in some movements in the three occlusal schemes (P<0.05) (Table 5).

**Table 5:** comparison the mean strain values in different areas in any movement in any occlusal schemes (με)

**Occlusion**	**Movement**	**(J) Area**	**(I) Area**	**Mean Difference (I-J)**	**Std. Error**	**P-value**
**Anatomic**	**Non-working**	Buccal shelf	Distolingual	-3.43750	1.24651	.030
		Mental foramen	Distolingual	-1.38750	1.24651	.517
		Mental foramen	Buccal shelf	2.05000	1.24651	.250
	**working**	Buccal shelf	Distolingual	-10.57500	1.36989	<.001
		Mental foramen	Distolingual	3.23333	1.36989	.061
		Mental foramen	Buccal shelf	13.80833	1.36989	<.001
**Lingualized**	**Protrusive**	Buccal shelf	Distolingual	-1.92500	.70278	.026
		Mental foramen	Distolingual	4.01667	.70278	<.001
		Mental foramen	Buccal shelf	5.94167	.70278	<.001
	**Non-working**	Buccal shelf	Distolingual	-1.81250	.12332	<.001
		Mental foramen	Distolingual	-3.41250	.12332	<.001
		Mental foramen	Buccal shelf	-1.60000	.12332	<.001
**Monoplane**	**Protrusive**	Buccal shelf	Distolingual	-.89167	.38014	.063
		Mental foramen	Distolingual	.35000	.38014	.631
		Mental foramen	Buccal shelf	1.24167	.38014	.007
	**Non-working**	Buccal shelf	Distolingual	-.57500	.90219	.803
		Mental foramen	Distolingual	-2.17500	.78132	.039
		Mental foramen	Buccal shelf	-1.60000	.78132	.140
	**Working**	Buccal shelf	Distolingual	-3.15833	.92952	.005
		Mental foramen	Distolingual	-.27500	.92952	.953
		Mental foramen	Buccal shelf	2.88333	.92952	.011

Table 5 showed that in the anatomic occlusal scheme on the working side, higher values were recorded in the buccal shelf than in the distolingual area and mental foramen (P<0.01 and P<0.01, respectively); however, no statistically significant difference was found between the distolingual area and mental foramen (P=0.061).

In lingualized occlusal scheme, during protrusive movement, a higher value was recorded in the buccal shelf area compared with distolingual area and mental foramen (P=0.026 and P<0.001, respectively). In addition, the value was higher in distolingual area compared with the mental foramen (P<0.001).

In the monoplane occlusal scheme in protrusion, the values were higher in the buccal shelf than the mental foramen (P=0.007).

On the non-working side, values were higher in ​​the mental foramen than the distolingual area (P=0.039). On the working side, values were higher in the buccal shelf than the distolingual area (P=0.005) and mental foramen (P=0.011), but there was no statistically significant difference between the mental foramen and the distolingual area (P=0.953).

## Discussion

The application of strain-gauge in dental research is one of the techniques used to evaluate biomechanical loads. This method is based on electrical resistance in strain gauges. It provides both in vitro and in vivo strain measurements under static and dynamic loads; it also provides data at definitive points. This method was very close to our aims.^17^

Transfer of chewing forces to the inferior edentulous ridge is influenced by the size, shape and occlusal schemes of denture posterior teeth. Understanding these forces and the distribution scheme of stress in the bone beneath complete denture is one of the most important factors when fabricating complete dentures. Ignoring these factors can lead to discomfort of denture wearer and resorption of the remaining alveolar ridge. Therefore, choosing an appropriate occlusal scheme is one of the most important factors in construction of complete dentures. In the present study, the distribution of strain in different areas of mandibular edentulous atrophied ridge was analyzed under the influence of different occlusal schemes during different jaw movements.

In this study, to compare strain exerted on the remaining ridge, a strain gauge was embedded on each side of the mandible in ​​the mental foramen, buccal shelf and distolingual areas in a jaw model prepared with epoxy resin. To simulate the alveolar mucosa over the resin cast, Gingifast (Zhermack, A-silicone for gingival mask, Italy) was used. Three groups of artificial teeth were set up based on their occlusal schemes. In order to provide similar test conditions, a resin model was used in all the three models, and the conditions of mounting, vertical height, and centric and eccentric relationships were similar in all the three states. Finally, after processing the dentures, they were mounted on a jaw movement simulator. It can induce the articulator to make lateral and protrusive movements at a range of 3 mm, and simultaneously, can exert a perpendicular force of 110 N on the occlusal surfaces of the teeth. The strain was recorded in above-mentioned conditions under the force of 110 N. According to Prombonas and Vlissidis study, the maximum bite force exerted by the edentulous patients at the vertical dimensional of occlusion is 110 N‏.^18^

According to the results of this study, when the three occlusal schemes were compared, the mean strain in the anatomical occlusal scheme was higher and in the monoplane occlusal scheme was lower than the two other schemes. In a study by Swoope and Kydd,^10^ reduction of cusp angle of posterior artificial teeth led to a decrease in pressure on complete denture bases.  Sharry et al^14^ showed on dry skull that more stress is exerted on the bone by anatomical teeth of the denture compared to the zero-degree teeth. Another study by Madalli et al^3^ compared the pressure on the denture-supporting area in different occlusal schemes concluded that the stress on the denture supporting area was lower in monoplane occlusal scheme than the anatomical and lingualized schemes.

In a study by Chowdhury et al,^19^ in comparison with 0-degree teeth greater magnitude of Stresses was observed in cuspal teeth, i.e. 33 and 20, respectively.

Lopuck et al reported that flat occlusal scheme transmitted slightly less force to the ridge than cuspal forms.^20^ Based on these studies we concluded that a change in the angulation of cusps might change the magnitude and direction of forces, and monoplane occlusion with zero-degree cuspal angulation exerted lower forces on mandibular ridge. Therefore, it is suitable for patients with parafunctional habits.^15^

Based on our study, the mean strain in the buccal shelf was generally higher than that in mental foramen and distolingual areas but no statistically significant difference existed between mental foramen and distolingual areas. This result supports the theory that the buccal shelf is the primary stress-bearing area.^15^ Meanwhile, according to a study by Madalli et al,^3^ in which the pressure on denture-supporting area was compared in different occlusal schemes, the pressure on ​​the buccal slope of mandibular ridge in the molar region (buccal shelf) was higher than other areas.

Based on the results of this study, the level of strain in all the three occlusal schemes was higher in eccentric movement on the working side compared with the non-working side, consistent with a study by Madalli et al,^3^ who showed that little pressure is exerted on the buccal slope of maxillary ridge on the non-working side in all the three occlusal schemes. Also, Frechette et al^21^ evaluated the distribution of chewing forces in artificial denture base in balanced and non-balanced occlusion and reported that the pressure in the remaining ridge on the working side increased 30‒80% in one-sided movements. In addition, the number of positive pressure strikes on the non-working side during one-sided mastication decreased, consistent with the results of the present study.

Based on the results of this study, the mean strain in anatomic occlusal scheme was higher in centric position compared with protrusive state, but in monoplane and lingualized occlusal schemes, it was higher in protrusive state than in centric position. It seems that the reason for this is the flatness of mandibular teeth in monoplane and lingualized occlusal schemes and cuspal state in anatomical occlusal scheme. In fact, in anatomical scheme lower force is exerted on ridge during exit from the centric position, due to the loss of the cusp fossa posture, but regarding monoplane and lingualized states, the cusp fossa contact is higher in protrusive movement, but no similar study was found for comparison.

Regarding the effect of studied areas on the mean strain variable in different movements, it was concluded that there was no statistically significant difference between the mean strain variable ​​in the studied areas in the static position, but in lateral movement, on the working side the mean strain was higher in the buccal shelf than ​​in the mental foramen and distolingual areas; In addition, in protrusive movement the mean strain was higher in the buccal shelf than ​​in mental foramen and distolingual areas. The results in relation to the lateral movements are similar to those of Madalli et al,^3^ where the pressure on ​​the buccal slope of the ridge (buccal shelf) was higher on the working side compared with the lingual slope of the ridge (distolingual) on the same side. Regarding protrusive movements, no similar study was found, but it seems that since the buccal shelf is the primary stress-bearing area, the mean strain in this area is higher than the mental foramen and distolingual areas.

On the other hand, the mean strain in protrusive movements was higher in the distolingual area than the mental foramen area, but in lateral movements, on the non-working side it was higher in ​​the mental foramen than in the distolingual areas. However, no similar study was found for comparison, but it seems that since in protrusive movements, the denture has an anteroposterior movement in the area, and distolingual area has a considerable impact on provision of retention, this denture area bears more strain during protrusive movement than the mental foramen area. But on the non-working side during lateral movements, strain increased in the mental foramen area compared with the distolingual area due to medio-lateral movement and contact on the ridge crest.

After investigating the effect of occlusal schemes and various movements on the mean strain in the studied areas, we concluded that during eccentric movements of the working side in monoplane and anatomical occlusal schemes, strain in the buccal shelf was higher than that in the distolingual area and mental foramen, consistent with the results of a study by Madalli et al,^3^ in which the pressure on the ​​buccal slope of the ridge (buccal shelf) in the monoplane and anatomical occlusal schemes on the working side was higher than that on the lingual slope of the ridge (distolingual) on the same side.

In lingualized occlusal scheme, the mean strain in protrusive movements in the buccal shelf was higher than that in the distolingual area and mental foramen, confirming that the buccal shelf is the primary stress-bearing area.8 However, comparison of distolingual area and mental foramen showed that strain was higher in the distolingual area than the mental foramen, and this result seems logical due to anteroposterior movement of denture and retention provided by the distolingual area.

## Conclusion

Despite the limitations in our study, the following results were obtained:

1. In general, the mean strain was higher in anatomical occlusal scheme and lower in monoplane occlusal scheme compared with the two other schemes. Therefore, monoplane occlusion is suitable for patients with residual ridge resorption and parafunctional habits.

2. In general and regardless of occlusal schemes, the mean strain in the buccal shelf area was higher than that in the mental foramen and distolingual areas but no statistically significant difference existed between the mental foramen and distolingual areas.

3. The mean strain in all the three occlusal schemes was higher on the working side than on the non-working side during eccentric movements. To achieve bilateral balance in the denture for providing stability it is better to pay attention to the number of contact areas on the non-working side. Therefore, we have broad stress distribution on both sides of the residual ridge and resorption of ridge will be reduced.

4. In the three occlusal schemes, the buccal shelf was the area bearing the highest force during different lateral, protrusive and centric movements. By knowing strain in the above-mentioned areas we can choose better techniques to achieve better stress distribution in mandibular denture-bearing areas.

## Acknowledgments

The authors would like to thank the Mechanics Laboratory of Tabriz University for assistance provided during the experiments of this study.

## Authors’ contributions

AH was the supervisor and designed the study. RK contributed to writing the manuscript and English editing. SGN contributed to the publication of the article and developing the protocol. MHB and MH contributed to the development of the protocol. NY contributed to the thesis, article writing, publication and data analysis.

## Funding

This study was supported by Dental and Periodontal Research Center, Faculty of Dentistry, Tabriz University of Medical Sciences, Tabriz, Iran.

## Competing interests

The authors declare no competing interests with regards to the authorship and/or publication of this article.

## Ethics approval

The study protocol was approved by the Ethics Committee of Tabriz University of Medical Sciences (code TBZMED.REC.1394.957).
